# Supplementary material for: A community-based validation of the International Alliance for the Control of Scabies Consensus Criteria by expert and non-expert examiners in Liberia
Source: PLoS Negl Trop Dis. 2020 Oct 5;14(10):e0008717. doi: 10.1371/journal.pntd.0008717 (PMC7732067; doi:10.1371/journal.pntd.0008717)
Supplement: S1 Table — (DOCX) [file pntd.0008717.s002.docx]

**S1 Table: False positives by expert diagnosis for mid-level health workers (MLHWs)**

| Expert Diagnosis | MHW 1 | MLHW 2 | MLHW 3 | MLHW 4 | MLHW 5 | MLHW 6 |
| --- | --- | --- | --- | --- | --- | --- |
| No Rash |  | 2 | 13 | 5 | 3 | 5 |
| Atopic Dermatitis |  | 5 | 5 | 4 | 1 | 7 |
| Tinea Corporis |  | 4 | 4 | 4 | 2 | 7 |
| Lichen Simplex | 1 | 3 |  | 3 | 1 | 5 |
| Tinea Capitis |  | 3 | 4 | 3 |  | 2 |
| Folliculitis |  | 2 | 2 | 2 | 3 | 3 |
| Lichen Planus | 1 | 2 | 2 | 2 | 1 | 2 |
| Pityriasis versicolor |  | 1 | 1 | 4 | 1 | 3 |
| Follicular Eruption |  |  | 1 | 3 | 1 | 2 |
| Erythrasma | 1 | 1 | 2 |  | 1 |  |
| Chronic Leg Wound | 1 | 1 | 1 |  |  | 1 |
| Port Wine Stain |  |  | 1 | 1 | 1 | 1 |
| Molluscum contagiosum |  | 1 | 1 |  |  | 1 |
| Eczema |  |  | 1 | 1 |  | 1 |
| Psoriasis |  | 1 |  | 1 |  |  |
| Insect Bite |  |  |  | 1 |  | 1 |
| Tinea Pedis |  | 1 |  |  |  |  |
| Follicular Eczema |  | 1 |  |  |  |  |
| Acne |  |  | 1 |  |  |  |
| Seborrhoeic Dermatitis |  |  | 1 |  |  |  |
| Abscess |  |  | 1 |  |  |  |
| Zoster |  |  |  |  |  | 1 |
