# Supplementary material for: A community-based validation of the International Alliance for the Control of Scabies Consensus Criteria by expert and non-expert examiners in Liberia
Source: PLoS Negl Trop Dis. 2020 Oct 5;14(10):e0008717. doi: 10.1371/journal.pntd.0008717 (PMC7732067; doi:10.1371/journal.pntd.0008717)
Supplement: S2 Table — (DOCX) [file pntd.0008717.s003.docx]

**S2 Table**: **False positives by IACS category for MLHWs**

| **MLHW** | **MLHW IACS category** | | | | | **Total false positive diagnoses** |
| --- | --- | --- | --- | --- | --- | --- |
|  | **B1** | **B2** | **B3** | **C1** | **C2** |  |
| **1** | 0 | 0 | 2 | 2 | 0 | 4 |
| **2** | 3 | 0 | 6 | 7 | 10 | 26 |
| **3** | 0 | 0 | 18 | 4 | 18 | 40 |
| **4** | 3 | 0 | 12 | 8 | 11 | 34 |
| **5** | 0 | 0 | 5 | 6 | 4 | 15 |
| **6** | 1 | 0 | 21 | 6 | 13 | 41 |

­
