# Supplementary material for: A community-based validation of the International Alliance for the Control of Scabies Consensus Criteria by expert and non-expert examiners in Liberia
Source: PLoS Negl Trop Dis. 2020 Oct 5;14(10):e0008717. doi: 10.1371/journal.pntd.0008717 (PMC7732067; doi:10.1371/journal.pntd.0008717)
Supplement: S3 Table — (DOCX) [file pntd.0008717.s004.docx]

**S3 Table: Complete list of diagnoses made on examination by a dermatologist**

| Diagnosis | n (%) |
| --- | --- |
| Scabies | 46 (31.1%) |
| Tinea corporis | 15 (10.3%) |
| Atopic dermatitis/Eczema | 17 (XX%) |
| Lichen Simplex | 9 (6.2%) |
| Tinea capitis | 7 (4.8%) |
| Folliculitis | 6 (4.1%) |
| Follicular Eruption | 6 (4.1%) |
| Impetigo | 4 (2.8%) |
| Pityriasis versicolor | 4 (2.8%) |
| Acne | 3 (2.1%) |
| Molluscum contagiosum | 2 (1.4%) |
| Vitiligo | 2 (1.4%) |
|  |  |
| Erythrasma | 2 (1.4%) |
| Insect Bite Reaction | 2 (1.4%) |
| Lichen Planus | 2 (1.4%) |
| Contact dermatitis | 1 (0.7%) |
| Psoriasis | 1 (0.7%) |
| Seborrhoeic dermatitis | 1 (0.7%) |
| Tinea pedis | 1 (0.7%) |
| Bacterial Abcess | 1 (0.7%) |
| Chronic Wound | 1 (0.7%) |
| Demographism | 1 (0.7%) |
|  |  |
| Herpes Zoster | 1 (0.7%) |
| Keloid | 1 (0.7%) |
| Lipoma | 1 (0.7%) |
| Port Wine Stain | 1 (0.7%) |
| No skin problem | 19 (12.9%) |
